# Racial/ Ethnic and Gender Differences in Older Adults’ Chronic Stress Patterns

**DOI:** 10.1093/geroni/igab046.3303

**Published:** 2021-12-17

**Authors:** Kun Wang, Zainab Suntai

**Affiliations:** University of Alabama, Tuscaloosa, Alabama, United States

## Abstract

Chronic stress has been associated with several adverse psychological, physical, and cognitive outcomes. While there exists a baseline level of stress among all individuals, certain groups of people are at risk of developing chronic stress due to existing hardships and stressors. Black Indigenous and/or people of color (BIPOC) and women have historically experienced several inequities including higher rates of certain chronic illnesses, interpersonal discrimination, socioeconomic disparities, and several other adverse outcomes. In addition to stressors from racial/ethnic and gender identities, older adulthood is a major transitional period marked by changes in physical, emotional, and cognitive well-being, which have been shown to affect overall well-being and mental health. As such, this study aimed to examine the association between chronic stress and cognition among older adults, using the intersectionality of race and sex. Data from the 2016 Health and Retirement Study were used, resulting in a final sample of 6,015 adults aged 50 and older. Latent class analysis was used to determine chronic stress patterns by sex and race, and a three-step method was used to examine the effects of covariates on stress class memberships by race and sex subgroups. Results indicated that compared to White men, the high stress classes among White women, BIPOC men and BIPOC women contained more stressors. Interventions targeted towards the mitigation of chronic stress among older adults should consider how intersectional identities combine to create increased hardships and stressors.

